# Exponential distribution of total depressive symptom scores in relation to exponential latent trait and item threshold distributions: a simulation study

**DOI:** 10.1186/s13104-017-2937-6

**Published:** 2017-11-23

**Authors:** Shinichiro Tomitaka, Yohei Kawasaki, Kazuki Ide, Maiko Akutagawa, Hiroshi Yamada, Toshiaki A. Furukawa

**Affiliations:** 1 Department of Mental Health, Panasonic Health Center, Landic Building 3F, Nishishinbashi 3-8-3, Minato-ku, Tokyo, 105-0003 Japan; 2 Department of Drug Evaluation and Informatics, School of Pharmaceutical Sciences, University of Shizuoka, 52-1 Yada, Suruga-ku, Shizuoka, 422-8526 Japan; 3 Department of Health Promotion and Human Behavior, Department of Clinical Epidemiology, Kyoto University Graduate School of Medicine/School of Public Health, Yoshida Konoe-cho, Sakyo-ku, Kyoto, 606-8501 Japan; 4 Clinical Research Center, Chiba University Hospital, 1-8-1, Cho-ku, Chiba-shi, Chiba, 260-8677 Japan; 5 Department of Pharmacoepidemiology, Graduate School of Medicine and Public Health, Kyoto University, Yoshida Konoe-cho, Sakyo-ku, Kyoto, 606-8501 Japan; 6 Center for the Promotion of Interdisciplinary Education and Research, Kyoto University, Yoshida-honmachi, Sakyo-ku, Kyoto, 606-8501 Japan

**Keywords:** Depressive symptom, Latent trait, Simulation, Ordinal scale, Exponential distribution, CES-D, CIS-R

## Abstract

**Objectives:**

Total depressive symptom scores in the general population have been reported to follow an exponential distribution except at the lowest end of the range of scores. To verify the hypothesis that total depressive symptom scores follow the distribution of the latent trait, we performed a simulation study of depressive symptom scoring modeled after the Revised Clinical Interview Schedule (CIS-R). To simulate the scoring of ordinal scale items in CIS-R, two sets of random numbers were generated, one expressing the degree of the latent trait of depressive symptoms and another expressing the threshold for each item. Random latent trait numbers greater than those of item thresholds indicated the presence of specific symptoms.

**Results:**

When exponential distribution was set to the latent trait’s random numbers and each item’s threshold had a certain degree of standard deviation, simulated total depressive symptom scores showed a linear pattern except at the lowest end of scores with a log-normal scale. Our results suggest that total depressive symptom scores follow the distribution of the latent trait of depressive symptoms due to the property of ordinal scales, which is characterized by individual differences in the threshold of each item.

**Electronic supplementary material:**

The online version of this article (10.1186/s13104-017-2937-6) contains supplementary material, which is available to authorized users.

## Background

Major depression is the fourth leading cause of disability worldwide and contributes to decreased functioning, diminished quality of life, and increased mortality in millions of individuals across a wide range of educational and socioeconomic levels [1]. Given that the presence of depressive symptoms is closely linked with clinical levels of depression, there has been great interest in understanding the distribution of depressive symptoms in the general population [2, 3]. However, to the best of our knowledge, there have been few mathematical models on the distribution of depressive symptoms based on observed data.

Building mathematical models inductively is of great importance in the natural sciences, particularly in physics. In fact, physical phenomena are usually expressed using mathematical models. To date, physicists have observed a physical phenomenon, found a reproducible pattern in the observed data, and built a mathematical model inductively. However, in the field of psychology, inductive modelling is still not well developed. Compared to physical phenomena, it may be difficult to identify mathematical patterns in psychological phenomena. Instead, psychology researchers mainly analyze observed data using statistical models, most of which are deductive. It is notable that building a mathematical model based on observed data is different from analyzing observed data using statistical models. For example, although the number of factors can be determined using factor analysis, this statistical procedure presupposes a linear relationship between variables and the intervality of the scoring system, implying that factor analysis is distinct from building a mathematical model inductively.

The purpose of our project was to build a mathematical model of item scores and total scores on depression screening scales based on the information obtained from observed data. In developing a mathematical model inductively, it is first necessary to identify a reproducible pattern in the observed data. Recently, several studies with large sample sizes have revealed that total depressive symptom scores in the general population approximate an exponential pattern, except at the lowest end of the range of scores [4, 5]. In an analysis of data from nearly 10,000 non-psychotic respondents to the British National Household Psychiatric Morbidity Survey, Melzer et al. observed that an exponential curve provided the best fit for total depressive and neurotic symptom scores on the Revised Clinical Interview Schedule (CIS-R) [4]. The authors of the present study have similarly observed that the right tail of the distribution of total depressive symptom scores on the Center for Epidemiologic Studies Depression Scale (CES-D) follows an exponential curve, based on data from a national survey of the Japanese population including data from nearly 25,000 respondents [5]. A similar study involving a large sample of Japanese employees further supported the exponential pattern of CES-D scores, except at the lowest end of the scale [6]. Moreover, in our analysis of data from the National Survey of Midlife Development in the United States (MIDUS), we demonstrated that the total scores of the 6-item Kessler Screening Scale for Psychological Distress (K6) commonly exhibited exponential patterns, except at the lowest end of the scale [7].

According to the theory of measurement proposed by Stevens, a sum or a mean of depressive symptom scores is meaningless because each depressive symptom item is scored using an ordinal scale, where the differences between each item may not necessarily be quantifiable [8]. Nevertheless, large sample surveys have consistently shown that total depressive symptom scores calculated by summing the scores from individual items still tend to follow this exponential pattern except at the lowest end of the range of scores. From a clinical point of view, the finding that total depressive symptom scores follow an exponential pattern is noteworthy because it may enable us to estimate the number of individuals with each degree of depressive symptoms. It therefore becomes important to clarify the potential mechanisms that contribute to this exponential distribution and investigate the relationships among the variables involved.

**Fig. 1:**
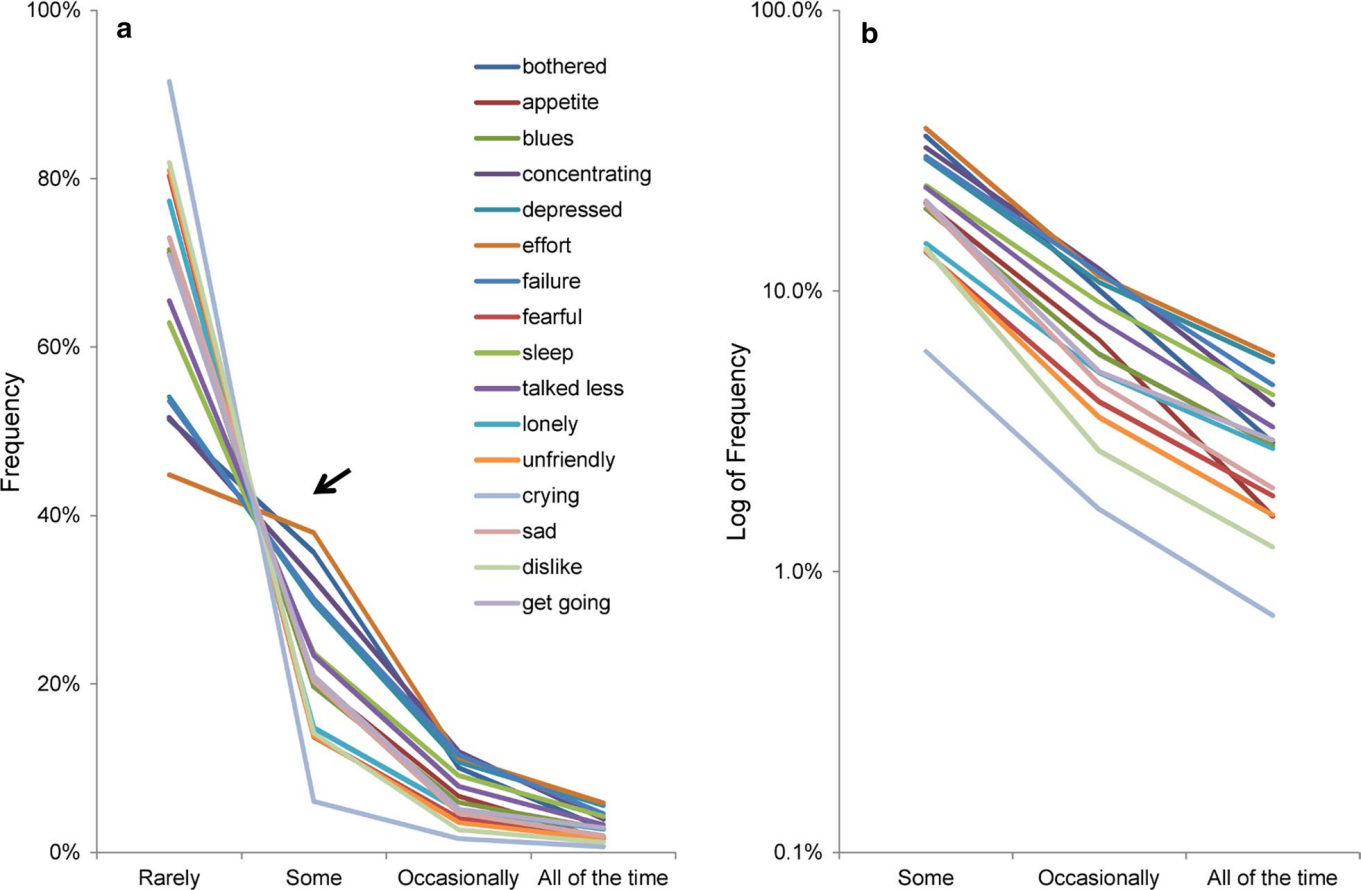
Item responses of the 16 depressive symptom items of the Center for Epidemiologic Studies Depression Scale on a normal and a log-normal scale. Item responses of the 16 items of depressive symptoms exhibited a common mathematical pattern on a normal scale (**a**) and a log-normal scale (**b**) [11]. http://dx.doi.org/10.1371/journal.pone.0165928.g001

Regarding the reproducible pattern of item responses in the general population, it was recently reported that the item responses on depression screening scales exhibited a common mathematical pattern among all items. The CES-D allows the individual to self-rate the frequency of a variety of depressive symptoms (sadness, fatigue, etc.) on a scale consisting of four possible responses: “rarely (less than 1 day)”, “some (1–2 days)”, “occasionally (3–4 days)”, and “all of the time (5–7 days)” [9]. We have recently demonstrated that, in a general population, responses to each of the 16 individual items related to negative symptoms of depression on the CES-D tend to exhibit exponential patterns between the “some” and “all of the time” responses, while this pattern is not observed for “rarely” responses [10, 11] (Fig. 1). These findings have also been reproduced in the same sample of Japanese employees for the CES-D and all four subsamples from the same MIDUS data for the K6 [6, 7].

Given that the mathematical pattern of item responses among all items and the exponential pattern of total scores were the same, we then proposed that all items related to depressive symptoms are manifest variables influenced by a unidimensional latent trait of depressive symptoms, which itself follows an exponential distribution [12, 13]. If the latent trait of each variable is not unidimensional, the distributions of the total scores will be determined by multiple latent traits. Thus, the total scores of a multiple latent model seem unlikely to converge to a unitary distributional pattern. From a neuroscientific standpoint, one latent trait is comprehensible. Many researchers have proposed that specific neural regions constitute a final common pathway underlying depressive symptoms [14, 15], suggesting that all depressive symptoms are influenced by a single latent trait.

In contrast to a normal distribution, an exponential distribution is a right skewed unimodal distribution. While a normal distribution emerges when many independent random variables are combined by addition, an exponential distribution emerges when individual variability and total stability are observed together, for example, in energy levels within atoms and individual income [16]. Although a common mathematical pattern of item responses was indeed observed, such a pattern would only be applicable in the case that the latent trait followed an exponential distribution. The previous study suggested that the finding that the boundary curves of each item score in the distribution of total scores approximate an exponential pattern is strongly related to the common mathematical pattern of item responses among all items [12].

The present study aims to clarify the potential mechanisms that contribute to the exponential pattern exhibited by total depressive symptom scores and to investigate the hypothesis that these total scores follow the distribution observed for the latent trait of depressive symptoms. The hypothesis is consistent with the fact that total scores on intelligence tests, which presuppose a normal distribution for the latent trait of intelligence, approximate a normal distribution [17, 18]. In order to verify the hypothesis that the total depressive symptom scores follow the exponentially distributed latent trait, it is also necessary to elucidate the impact of each item score when a unidimensional latent trait is followed. However, to the best of our knowledge, little work has been done to develop a model of ordinal scales for a unidimensional latent trait.

The present study assumes that the ordinal scales for depressive symptoms are characterized by individual differences in item rating: although all participants use the same criteria for each item related to depressive symptoms, each participant is assumed to exhibit an individual threshold for each depressive symptom. For example, participants with the same degree of the latent trait—depressive symptoms, in this case—may differ in the degree to which they are affected by sleep disturbances. In short, individual thresholds for each depressive symptom are expected to differ from each other, with each item exhibiting its own unique distribution. Meanwhile, the latent trait of depressive symptoms also differs according to the individual and constitutes an exponential distribution. When the degree of a participant’s latent trait is greater than that of the threshold for the specific depressive symptom, the specific symptom is expected to be present.

To ascertain the validity of the hypothesis that total depressive symptom scores follow a distribution similar to the exponential pattern observed for the latent trait, we performed a simulation study of depressive symptom scoring using the aforementioned ordinal scale model. The model comprises the following three conditions: (1) depressive symptom items are manifest variables influenced by a unidimensional latent trait, (2) the latent trait of depressive symptom items, which varies from individual to individual, forms an exponential distribution, and (3) the threshold of ordinal scale scoring varies from individual to individual, and forms a normal distribution in accordance with the unidimensional latent trait. Furthermore, to evaluate the effect of individual differences on each item’s threshold, we investigated the role of the standard deviation of each item’s threshold in generating the distribution of total scores depressive symptom scores using such a simulation study.

## Methods

### The model of an ordinal scale for depressive symptoms

The present study utilized the CIS-R as a model for the simulated rating scale of depressive symptoms [19]. The CIS-R is a psychiatric rating scale developed to measure levels of depressive and neurotic symptoms and contains 14 items related to negative symptoms: somatic symptoms, fatigue, concentration, sleep, irritability, worry about physical health, depression, depressive ideas, worry, anxiety, phobias, panic, compulsions, and obsessions. Each item on the CIS-R is evaluated using the participant’s responses to four associated questions scored using a binary method (0 or 1). Each question then contributes a single point on the 0–4 scale for each item (except depressive ideas, which has a 0–5 scale). Thus, the total score of CIS-R ranges from 0 to 57.

**Fig. 2:**
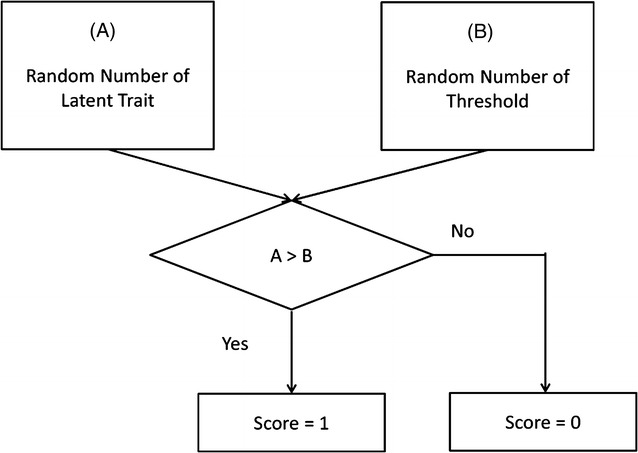
Process model of a binary scale of depressive symptoms. Two sets of random numbers, representing individuals with a specific degree of a depressive symptom’s latent trait and a specific threshold for each depressive symptom, are generated. If the random number of the depressive symptom’s latent trait is greater than that of the specific item’s threshold, the specific symptom is considered present

To simulate an ordinal scale for depressive symptoms, we used a process model in which depressive symptom scores were determined by two factors—the degree of a depressive symptom’s latent trait and the threshold for each depressive symptom (see Fig. 2). If the random number of the depressive symptom’s latent trait was greater than that of the specific depressive symptom’s threshold, the specific symptom was considered present (see Additional file 1).

### Distribution of latent trait

In line with our previous assumption that the latent trait of depressive symptoms followed an exponential distribution, the simulated latent trait was expressed as an exponentially distributed random number. As the real parameter λ of the latent trait’s exponential distribution was difficult to estimate, the parameters’ values were allocated as examples. Exponential distributions with λ values of 1, 2, and 3 were used in the simulation of the latent trait distribution.

### Distribution of each item’s threshold

In developing the model based on an ordinal scale, we assumed that each simulated participant had a threshold for each criterion related to depressive symptoms and that the threshold for each criterion form a distribution around the mean value. Thus, in the present simulation, each item’s threshold was set to a normal distribution, with each item being locally independent from the others.

To compare the latent trait’s random number with that of the threshold, each item’s threshold must share the scale with the latent trait. Therefore, the mean distribution value of each item’s threshold was set to the percentile point that corresponds with each item’s rate of prevalence (see Additional file 2). As a reference for prevalence rates for CIS-R items, we used data from the Adult Psychiatric Morbidity Study in England (APMS 2007) [20]. The sample for APMS 2007 was designed to represent the population aged 16 and over living in private households in England. Details of the APMS 2007 have been described elsewhere [20].

Specifically, the prevalence rate for “somatic symptom 1” (*I had pain or discomfort more than 4* *days in the past week*) was 5.49% according to the APMS 2007. The percentile point of 5.49% was 2.90 when the parameter of λ = 1 was set the exponential distribution of the latent trait of depressive symptoms (see Additional file 2). The value of 2.90 was allocated to the mean value that corresponded to the threshold distribution of somatic symptom 1.

Similarly, percentile points of the specific latent trait distribution that corresponded to prevalence rates of all 57 depressive symptoms were calculated; calculated percentile point values were then allocated to mean normal distribution values that corresponded to distributions of all 57 depressive symptom thresholds. Additional file 2: Table S1 shows the calculated percentile point values of prevalence rates for the 57 depressive symptoms. Values of 1, 2, and 3 were used for λ in the exponential distribution of the latent trait.

To investigate the relationship between the item’s threshold distribution and the distribution of total depressive symptoms scores, we set some conditions for the standard deviation of each item’s threshold and evaluated the mathematical distribution pattern of total depressive symptom scores. Each item’s threshold was set to follow a normal distribution with standard deviation values of 1, 2, 3, and 4.

After confirming that the simulated total depressive symptom scores more closely approximate the exponential distribution as the standard deviations of the thresholds increased, we investigated the distribution of total depressive symptom scores under the condition that thresholds of depressive symptoms were set to uniform distribution with parameters 0 and 5 (see Additional file 3).

### Analysis procedure

The simulated dataset had a sample size of 10,000, independently generated using SAS Version 9.4 for Windows (SAS Institute, Inc., Cary, NC, USA). The sample size of 10,000 was selected to approximate the number of subjects used in several population surveys of depression symptoms [4]. The mathematical distribution pattern of total depressive symptom scores was graphically analyzed using a normal scale and a log-normal scale.

## Results

**Fig. 3:**
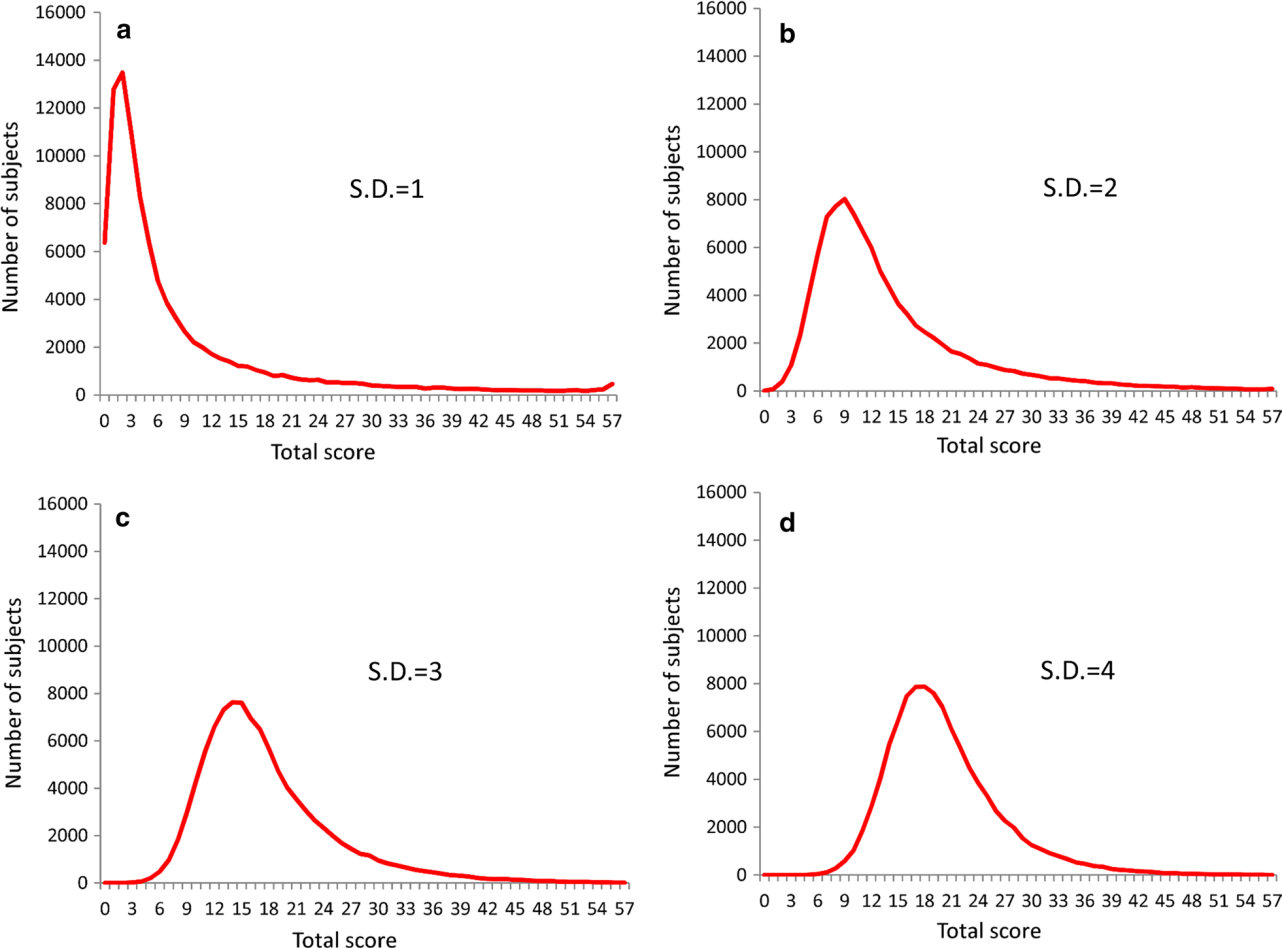
Distributions for simulated total depressive symptom scores when the latent trait was set to exponential distribution with a parameter of λ = 1. Exponential distribution with a parameter of λ = 1 was set for the latent trait of depressive symptoms, and normal distributions with standard deviations of 1 (**a**), 2 (**b**), 3 (**c**), and 4 (**d**) were used for the distribution of depressive symptom thresholds. *SD* standard deviations

Figure 3 depicts the distributions for the simulated total depressive symptom scores when the latent trait was set to an exponential distribution with a parameter of λ = 1. Total simulated depressive symptom scores exhibited right-skewed distribution, with the skewness of the distributions decreasing as the standard deviations of the threshold increase.

**Fig. 4:**
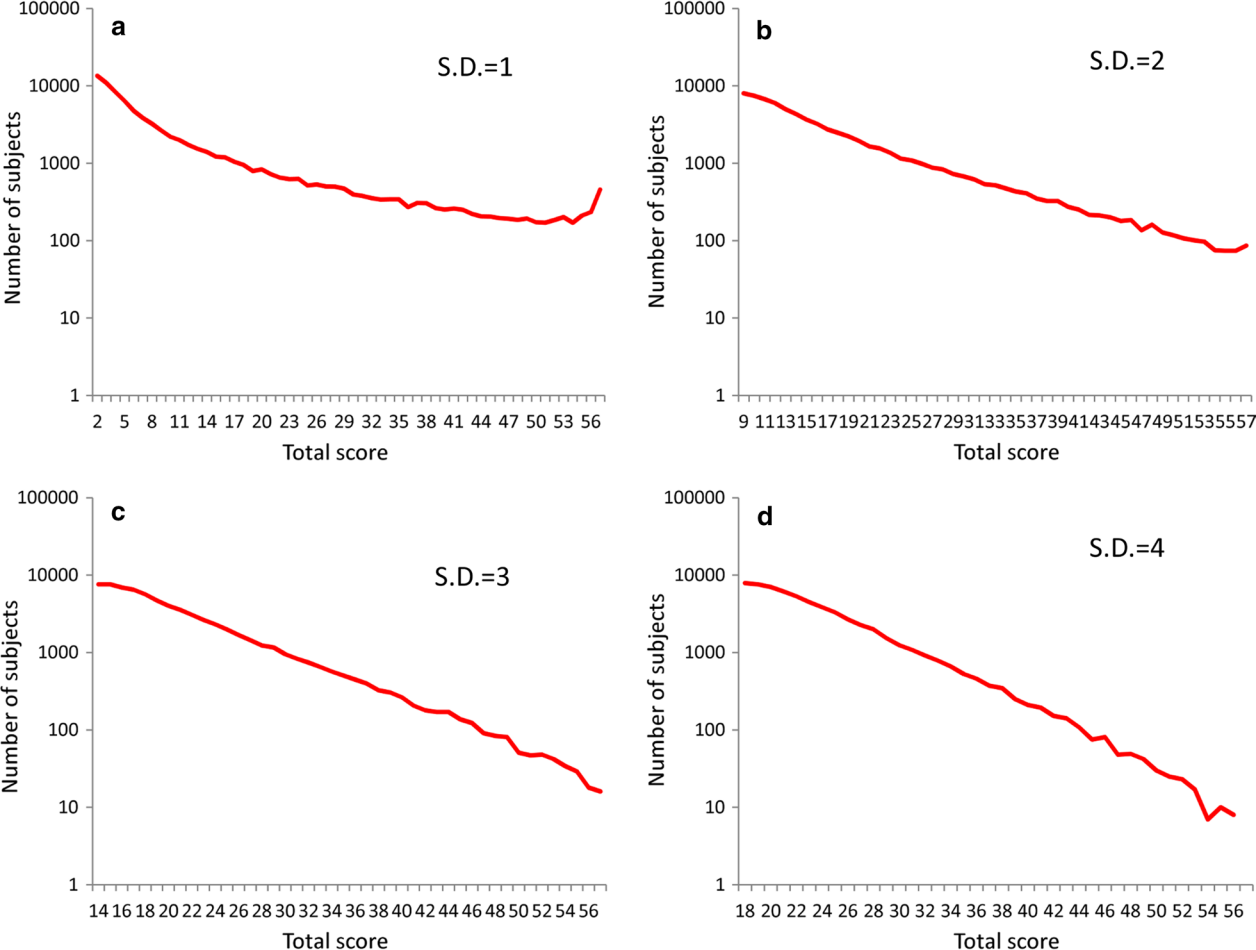
Distributions for simulated total depressive symptom scores with a log-normal scale when the latent trait was set to an exponential distribution with a parameter of λ = 1. Exponential distribution with a parameter of λ = 1 were used for the latent trait of depressive symptoms, and normal distributions with standard deviations of 1 (**a**), 2 (**b**), 3 (**c**), and 4 (**d**) were applied for the threshold of depressive symptom items

To demonstrate the mathematical patterns of the distributions of total depressive symptom scores, the right tails of total depressive symptom scores were evaluated using a log-normal scale (Fig. 4). When the standard deviations of each item’s threshold were set to values of 2, 3, and 4, the right tails of the distributions for total depressive symptom scores followed a linear pattern with a log-normal scale (see Fig. 4b–d); however, this linear pattern was not observed for standard deviation values of 1 (see Fig. 4a).

**Fig. 5:**
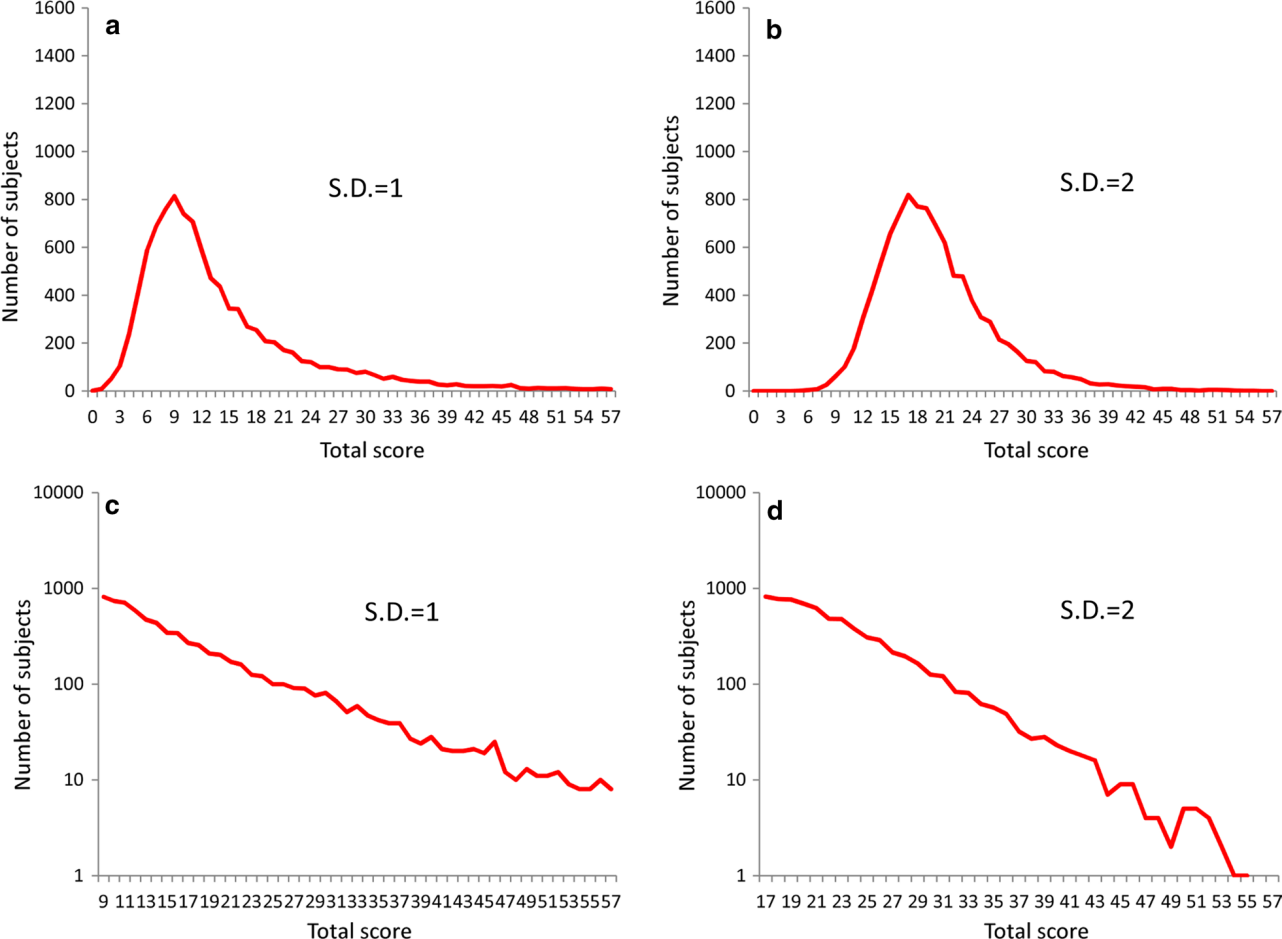
Distributions for simulated total depressive symptom scores when the depressive symptoms’ latent traits were set to exponential distributions with a parameter of λ = 2. Exponential distribution with a parameter of λ = 2 was used for the distribution of the latent trait, and normal distributions with standard deviations of 1 (**a**, **c**) and 2 (**b**, **d**) were applied to distribution of the threshold of depressive symptoms items. **a**, **b** With a normal scale and **c**, **d** are with a log-normal scale

Compared with those in which exponential distributions with the parameter of λ = 1 were set to the latent trait of depressive symptoms (see Fig. 3a, b), distributions of total depressive symptom scores with the parameter of λ = 2 set to the latent trait exhibited less skewed distribution (see Fig. 5a, b). Although the threshold distributions were set to the same normal distribution having equal standard deviations, the right tail of the total depressive symptom scores distribution, followed a linear pattern with a log-normal scale for λ = 2 (see Fig. 5c) but not for λ = 1 (see Fig. 4a).

**Fig. 6:**
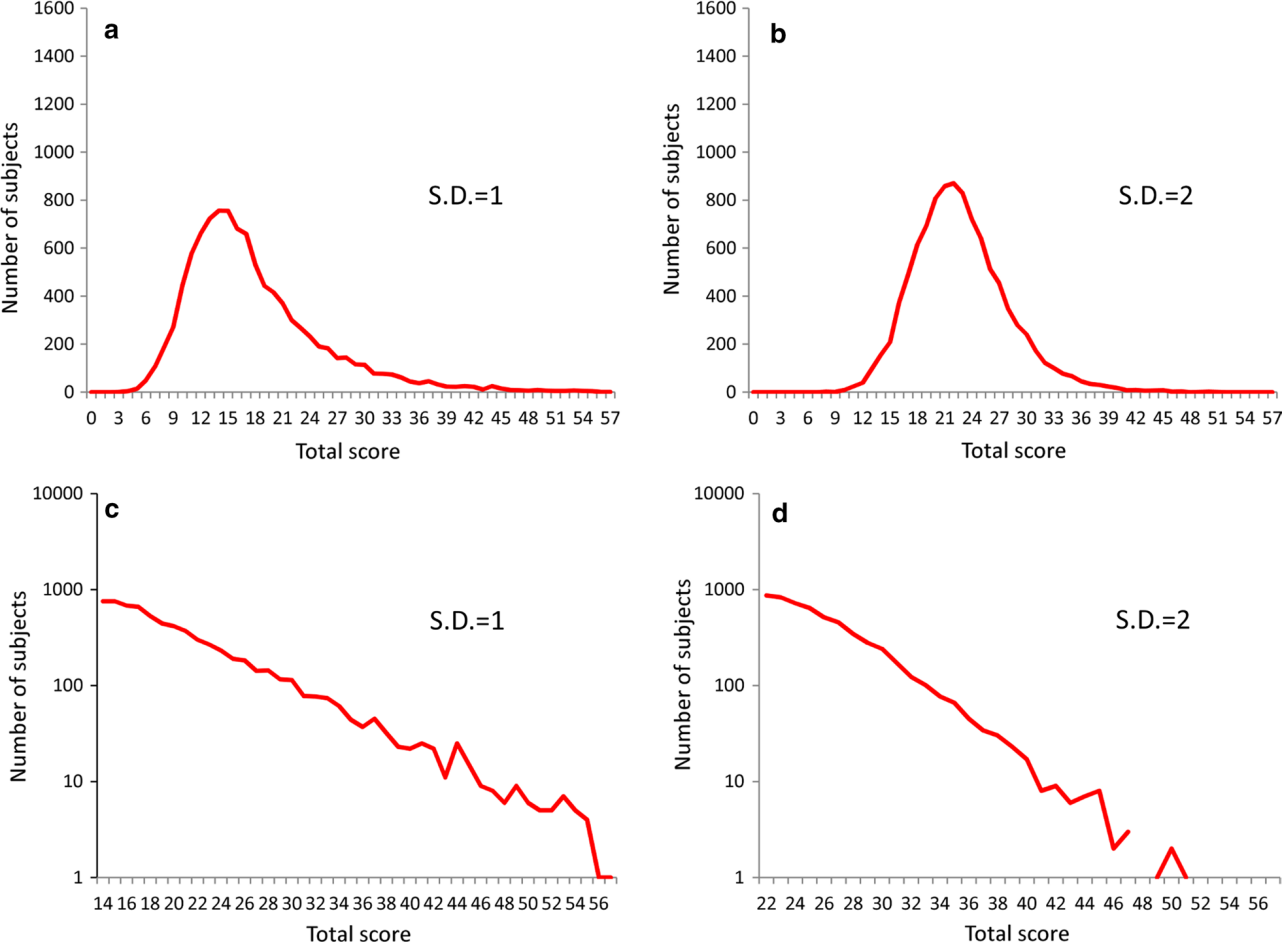
Distributions for simulated total depressive symptom scores when the latent trait was set to exponential distributions with a parameter of λ = 3. Exponential distribution with a parameter of λ = 3 was used for the latent trait of depressive symptoms, and normal distributions with standard deviations of 1 (**a**, **c**) and 2 (**b**, **d**) were applied to the distribution of the threshold of depressive symptom items. **a**, **b** With a normal scale and **c**, **d** are with a log-normal scale

When λ = 3 for the exponential distribution of the latent trait, total depressive symptom scores, with threshold standard deviation values of 1 and 2, also exhibited a linear pattern with a log-normal scale (see Fig. 6).

**Fig. 7:**
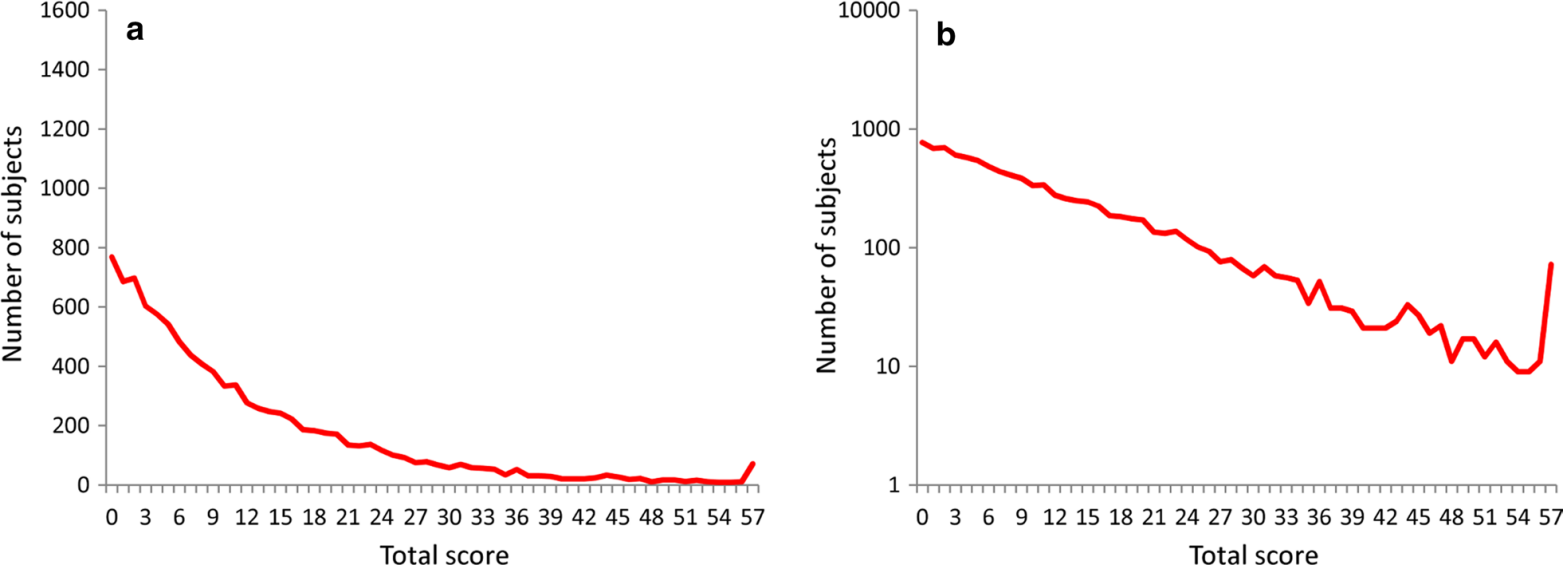
Distributions for simulated total manifest variable scores when each item’s threshold was set to uniform distribution. Exponential distribution with the parameter value of λ = 1 was used for the latent trait of depressive symptoms, and uniform distribution with parameters 0 and 5 was applied for the thresholds of manifest variables. **a** With a normal scale and **b** with a log-normal scale

To investigate the role of the item threshold standard deviations in the mathematical distribution of total depressive symptom scores, uniform distributions with parameters of 0 and 5 were used. When the latent trait followed an exponential distribution, and when threshold values were uniformly distributed, symptom scores exhibited a right-skewed distribution that lacked a left tail (see Fig. 7a). Furthermore, total depressive symptom scores exhibited a linear pattern in all ranges with a log-normal scale (see Fig. 7b).

## Discussion

The aim of present paper is to clarify the potential mechanisms that contribute to the exponential pattern of total depressive symptoms scores. We hypothesized that total depressive symptom scores approximate an exponential distribution because they follow the distribution of the latent trait. The results of the present study demonstrate that, when the latent trait was set to an exponential distribution and when each item’s threshold had a certain degree of standard deviation, the distribution of simulated total depressive symptom scores followed an exponential distribution except at the lowest end of the range of scores, confirming this initial hypothesis.

The findings that simulated total depressive symptom scores approximate the distribution of latent trait may shed light on the validity of total depressive symptom scores. In 1946, Stevens claimed that psychological measurement was an ordinal scale and that a sum or a mean of psychological measurement had no validity [8, 21, 22]. However, the results of the present study are inconsistent with this theory, instead revealing that the sum of ordinal scale variables could provide important information about latent trait of manifest variables.

At the lowest end of scores, the distribution of simulated total depressive symptom scores did not follow an exponential pattern. In recent studies, both Melzer et al. and our group have observed that, except at the lowest end of scores, total depressive symptom scores follow an exponential distribution in a general population [4, 5]. The results of the present simulation are consistent with those of population studies in that total depressive symptom scores do not follow an exponential distribution at the lowest end of scores. The range of the exponential pattern is clinically noteworthy because the range covers a high degree of depressive symptoms, which correspond to diagnoses of clinical depression [6]. Mathematically, an exponential distribution has a heavier tail than a normal distribution, suggesting that a considerable number of individuals with a high degree of depressive symptoms exist in a general population.

The results of the present study further support that a unidimensional latent trait model of depressive symptoms is suitable for the explanation of how total scores follow an exponential distribution. Most studies that utilize taxometric analysis—a statistical technique designed specifically to determine whether a given construct is best conceptualized by two discrete latent categories or one continuous latent dimension—support a unidimensional latent trait of depressive symptoms [23, 24]. Several lines of evidence of the unidimensional latent trait raise questions regarding the current categorical classifications of major depression and other associated disorders [25]. Although we use the term “latent trait”, the meaning of the term differs from that in taxometric analysis. We hypothesize that manifest variables follow the unidimensional latent trait because item responses exhibit the same pattern among all items (Fig. 1). Our latent trait is an inductive hypothesis based on the item response pattern and the latent trait is single. Conversely, taxometric analysis presupposes a statistical model and determine whether a given data is best conceptualized by two discrete latent categories or one continuous latent dimension. Thus, the latent trait of taxometric analysis is a deductive approach and the number of latent traits is determined according to the model fitness.

Our results indicate that a certain degree of standard for deviation for each item’s threshold is necessary to cause the simulated total depressive symptom scores to follow the exponential pattern. Assuming that ordinal scales for depressive symptoms are inherently characterized by individual differences in the threshold for particular items, the variance in each item’s threshold could be interpreted as a natural property of ordinal scales. Our results suggest that total depressive symptom scores follow the distribution of the latent trait due to this property.

### Total depressive symptom scores following an exponential distribution regardless of the scoring method

The present study utilized the CIS-R, which employs a binary method (0–1) of scoring, as a model for the depressive symptom rating scale [19]. We previously reported that total depressive symptom scores based on the CES-D and K6, which instead uses a four-point Likert scale and five-point Likert scale respectively, follow an exponential distribution except at the lowest end of scores [5, 7]. Regardless of the scoring method used, however, it is still necessary to consider why the total depressive symptom scores follow an exponential distribution. In the CES-D, each item has one question that is scored using a four-point Likert method (0–1–2–3) based on four possible answers: “rarely”, “some”, “occasionally”, and “all of the time”. Since adjacent answers are divided by a threshold, there are three thresholds for each item question [26]. If the 16 negative symptom items are manifest variables influenced by a unidimensional latent trait of depressive symptoms, the CES-D has 48 thresholds within a unidimensional latent trait. On the other hand, the CIS-R has 57 thresholds within a unidimensional latent trait. Since both the CIS-R and CES-D are structured in the same way, total depressive symptom scores would follow an exponential distribution regardless of the scoring method used for each item. Theoretically, total depressive symptom scores will also follow an exponential distribution regardless of the number of scale points.

The reason that the latent trait of depressive symptoms follows such an exponential pattern is unknown. As noted in the Introduction, exponential distribution is observed in populations in which individual variability and total stability are organized together, such as in the Boltzmann–Gibbs law and the principle of maximum entropy [16, 27]. With respect to individual variability and total stability, conditions that enable exponential distribution could be present in depressive symptoms [5, 13, 28].

### Mechanism behind total depressive symptom scores following the latent trait distribution

In the present study, as the degree of standard deviations for the thresholds increased, the total depressive symptom scores approached the latent trait’s distribution pattern. In addition, total depressive symptom scores exhibited a definite exponential pattern in all ranges when each item’s threshold was set to uniform distribution. These findings suggest that the key to understanding the mechanism behind total depressive symptom scores following the distribution of the latent trait lies in the magnitude of the thresholds’ standard deviations.

Consistent with this assumption, even though the same standard deviation value of 1 was set for each item’s threshold, total depressive symptom scores, when λ = 2 for the latent trait distribution, showed a linear pattern with a log-normal scale (see Fig. 5c); whereas, with a parameter of λ = 1 they did not (see Fig. 4a). Mathematically, the standard deviation of an exponential distribution with a parameter of λ is 1/λ.

In general, as the magnitude of the standard deviation of a normal distribution increases, normal distribution approaches uniform distribution. One must then consider why total depressive symptom scores follow the latent trait distribution when the item thresholds follow uniform distribution. When each item’s threshold follows uniform distribution, the threshold itself becomes meaningless since each item’s score is determined at a constant ratio regardless of the degree of the latent trait. Thus, the frequency of positively scored subjects of each item commonly follows the latent trait distribution. Eventually, total depressive symptom scores could follow the latent trait distribution. Further explanations using numerical expressions are necessary.

### Strengths and limitations

The present study has several limitations. First, in the simulation for latent trait distribution, we covered only exponential distribution. Thus, we have not ruled out the possibility that other mathematical distributions of latent traits might cause total depressive symptoms scores to express an exponential distribution. Further simulation studies are necessary to conclude that the distribution of the composite score of the manifest variables follows the latent trait distribution.

Second, given that a certain degree of standard deviations for thresholds of depressive symptoms is necessary to cause total depressive symptom scores to express the latent trait’s distribution pattern, it is informative to analyze CIS-R’s raw data to estimate the degree of standard deviations for depressive symptoms’ thresholds. However, we do not have the authority to access CIS-R raw data from large surveys (e.g., British National Survey of Psychiatric Morbidity [19]).

Despite these limitations, the present study provides important information regarding the theory of depressive symptoms scale. The degree to which present findings can be generalized to empirical data is unclear, though further examination is warranted.

## Conclusions

Our results indicate that total depressive symptom scores follow the distribution of the latent trait of depressive symptoms on the condition that each item’s threshold distribution has a certain degree of standard deviation. Furthermore, these results raise the possibility that the distribution type of the latent trait maybe elucidated by analyzing the distribution pattern of manifest variables’ total scores.

## Additional files

**Additional file 1.** SAS codes for the present simulation.

**Additional file 2.** The percentile point corresponding to the prevalence rate of each depressive symptom. As a reference for the prevalence rates of depressive symptoms surveyed with CIS-R, we used data of APMS 2007.

**Additional file 3.** Raw data for all figures.

## Abbreviations

APMS: Adult Psychiatric Morbidity Study in England
CES-D: Center for Epidemiologic Studies Depression Scale
CIS-R: Revised Clinical Interview Schedule
K6: 6-item Kessler Screening Scale for Psychological Distress
MIDUS: National Survey of Midlife Development in the United States
