# Supplementary material for: Exponential distribution of total depressive symptom scores in relation to exponential latent trait and item threshold distributions: a simulation study
Source: BMC Res Notes. 2017 Nov 23;10:614. doi: 10.1186/s13104-017-2937-6 (PMC5701434; doi:10.1186/s13104-017-2937-6)
Supplement: Supplementary file 2 — Additional file 2. The percentile point corresponding to the prevalence rate of each depressive symptom. As a reference for the prevalence rates of depressive symptoms surveyed with CIS-R, we used data of APMS 2007. [file 13104_2017_2937_MOESM2_ESM.docx]

**Supplementary Table 1. The percentile point corresponding to the prevalence rate of each depressive symptom.** As a reference for the prevalence rates of depressive symptoms surveyed with CIS-R, we used data of Adult Psychiatric Morbidity in England (APMS 2007).

| Depressive symptom | Prevalence  rate (%) | Percentile point | | |
| --- | --- | --- | --- | --- |
|  |  | Exponential  distribution  (λ＝1) | Exponential  distribution  (λ＝2) | Exponential  distribution  (λ＝3) |
| somatic 1 | 5.50 | 2.90 | 1.45 | 0.97 |
| somatic 2 | 6.15 | 2.79 | 1.39 | 0.93 |
| somatic 3 | 3.67 | 3.30 | 1.65 | 1.10 |
| somatic 4 | 5.34 | 2.93 | 1.47 | 0.98 |
| fatigue 1 | 25.03 | 1.39 | 0.69 | 0.46 |
| fatigue 2 | 24.57 | 1.40 | 0.70 | 0.47 |
| fatigue 3 | 26.64 | 1.32 | 0.66 | 0.44 |
| fatigue 4 | 21.96 | 1.52 | 0.76 | 0.51 |
| concentration 1 | 12.08 | 2.11 | 1.06 | 0.70 |
| concentration 2 | 10.28 | 2.28 | 1.14 | 0.76 |
| concentration 3 | 8.16 | 2.51 | 1.25 | 0.84 |
| concentration 4 | 6.81 | 2.69 | 1.34 | 0.90 |
| sleep1 | 21.10 | 1.56 | 0.78 | 0.52 |
| sleep2 | 35.48 | 1.04 | 0.52 | 0.35 |
| sleep3 | 27.12 | 1.30 | 0.65 | 0.43 |
| sleep4 | 9.52 | 2.35 | 1.18 | 0.78 |
| irriability1 | 10.55 | 2.22 | 1.11 | 0.75 |
| irriability2 | 12.56 | 2.07 | 1.04 | 0.69 |
| irritability3 | 25.76 | 1.36 | 0.68 | 0.45 |
| irritability4 | 18.47 | 1.69 | 0.84 | 0.56 |
| worry over physical health1 | 11.40 | 2.17 | 1.09 | 0.72 |
| worry over physical health 2 | 9.51 | 2.35 | 1.18 | 0.78 |
| worry over physical health3 | 3.81 | 3.27 | 1.66 | 1.09 |
| worry over physical health4 | 2.39 | 3.73 | 1.89 | 1.24 |
| depression1 | 11.98 | 2.12 | 1.06 | 0.71 |
| depression2 | 10.44 | 2.26 | 1.30 | 0.75 |
| depression3 | 12.35 | 2.09 | 1.05 | 0.70 |
| depression4 | 4.70 | 3.06 | 1.53 | 1.08 |
| depressive ideas1 | 9.54 | 2.35 | 1.18 | 0.78 |
| depressive ideas2 | 9.27 | 2.38 | 1.19 | 0.79 |
| depressive ideas3 | 9.33 | 2.37 | 1.19 | 0.79 |
| depressive ideas4 | 1.61 | 1.83 | 0.91 | 0.61 |
| depressive ideas5 | 0.59 | 2.82 | 1.41 | 0.94 |
| worry1 | 19.40 | 1.64 | 0.82 | 0.55 |
| worry2 | 22.63 | 1.49 | 0.74 | 0.05 |
| worry3 | 8.24 | 2.50 | 1.25 | 0.83 |
| worry9 | 14.20 | 1.95 | 0.98 | 0.65 |
| anxiety1 | 4.80 | 3.04 | 1.52 | 1.01 |
| anxiety2 | 4.44 | 3.11 | 1.56 | 1.04 |
| anxiety3 | 13.32 | 2.02 | 1.01 | 0.67 |
| anxiety4 | 5.58 | 2.89 | 1.44 | 0.96 |
| phobias1 | 2.78 | 3.58 | 1.79 | 1.19 |
| phobias2 | 8.23 | 2.50 | 1.25 | 0.83 |
| phobias3 | 7.06 | 2.65 | 1.33 | 0.88 |
| phobias4 | 2.08 | 3.87 | 1.94 | 1.29 |
| panic1 | 3.27 | 3.42 | 1.71 | 1.14 |
| panic2 | 1.38 | 4.28 | 2.14 | 1.43 |
| panic3 | 1.99 | 3.92 | 1.96 | 1.31 |
| panic4 | 2.16 | 3.83 | 1.92 | 1.28 |
| compulsion1 | 3.28 | 3.42 | 1.71 | 1.14 |
| compulsion2 | 2.81 | 3.57 | 1.79 | 1.19 |
| compulsion3 | 2.55 | 3.67 | 1.83 | 1.22 |
| compulsion4 | 3.46 | 3.36 | 1.68 | 1.12 |
| obsessions1 | 3.44 | 3.37 | 1.68 | 1.12 |
| obsessions2 | 5.31 | 2.94 | 1.47 | 0.98 |
| obsessions3 | 4.15 | 3.18 | 1.59 | 1.06 |
| obsessions4 | 4.12 | 3.19 | 1.59 | 1.06 |
